# Methodology for Selecting the Appropriate Electric Motor for Robotic Modular Systems for Lower Extremities

**DOI:** 10.3390/healthcare10102054

**Published:** 2022-10-17

**Authors:** Dimitrios Kavalieros, Evangelos Kapothanasis, Athanasios Kakarountas, Thanasis Loukopoulos

**Affiliations:** Department of Computer Science and Biomedical Informatics, University of Thessaly, 35131 Lamia, Greece

**Keywords:** robotic exoskeletons, servo motor, brushless, brushed, actuators, torque, centre mass

## Abstract

Torque calculation is essential for selecting the appropriate motor to achieve the required torque at each joint of a hybrid exoskeleton. In recent years, the combined use of functional electrical stimulation (FES) and robotic devices, called hybrid robotic rehabilitation systems, has emerged as a promising approach for the rehabilitating of lower limb motor functions. Specifically, the implementation strategy of functional electrical stimulation walking aid combined with the design of the exoskeleton part is the main focus of our research team. This work copes with issues of the design process of a robotic exoskeleton. The importance of robotic exoskeletons for providing walking aid to people with mobility disorders or the elderly is discussed. Furthermore, the approaches to calculating the joint torques are investigated, and the mathematical models and parameters of interest are identified. This further includes the comparative data for servo motors: robotic exoskeleton characteristics and actuator analysis in the robotic exoskeleton. The aforementioned is used to propose a mathematical model based on previous models (Zatsiorsky BSP and Dempster BSP body segment parameters models, forward kinematics models), which was extended to include added adjustable parameters such as length, area, volume, mass, density, the centre of mass, human body characteristics, and considering both static and dynamic parameter extraction. Then, an analytic method is presented, exploiting the results from the mathematical model to select the appropriate motor for each joint of the lower extremities. The detailed description of the method is followed by examples, experimental measurements, and statistical analysis of qualitative and quantitative characteristics. The results showed deviations from typical calculation methods, offering a better understanding of the motor requirements for each joint of the exoskeleton and avoiding selections of marginal functionality features of the motors. In addition, researchers are offered a tool for replicating the results of this work, allowing them to configure the parameters associated with the servo motor features. The researcher can either use the embedded library developed for this work or enter new data into it, affecting the calculated torques of the model joints. The extracted results assist the researcher in choosing the appropriate motor among commercially available brushed and brushless motors based on the torques applied at each joint in robotic articulated systems.

## 1. Introduction

Lately, exoskeletons are designed to provide strength in gait and heavy transport loads. There are also designs for assisting people with disorders in motion or older adults. Gait rehabilitation is one of the most significant challenges for society in the coming years due to population ageing and the increase of diseases affecting motion. Partial or total paralysis of one side of the body due to injuries in the motor centres of the brain is called Hemiplegia. Hemiplegia is a disorder that causes one-half of the human body to fail to perform its functions. This disorder is caused mainly due to stroke, and in many cases, it is hereditary. Recovery from a stroke is complex, and the treatment is prolonged. Wearable robotics is an area that provides solutions for such problems. A wearable robot extends, complements, or empowers the human limb where it is worn. These kinds of robots are classified according to the function they perform:

- **Empowering robotic exoskeletons:** These kinds of robots are known as extenders since they extend the strength of the human hand beyond its natural ability while maintaining human control of the robot.
- **Orthotic robots:** An orthosis maps the anatomy of a limb to restore lost functions. The robotic counterpart of orthosis is robotic exoskeletons that complement the ability of the limbs. Exoskeletons are also capable of restoring handicapped functions.
- **Prosthetic robots:** These robots are devices that fully substitute lost limbs [1].

**Figure 1:**
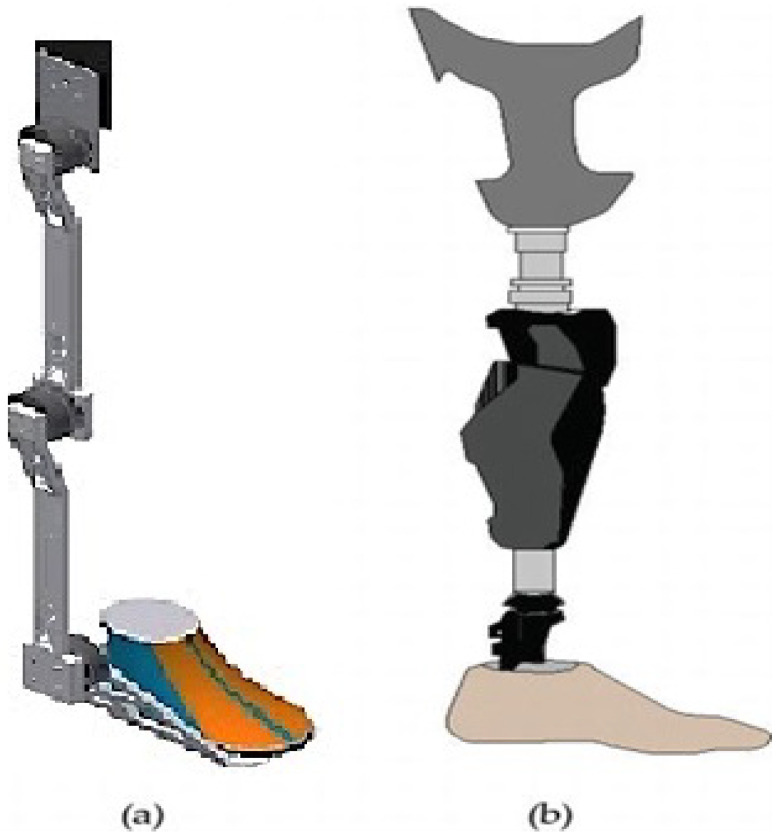
(**a**) Lower limb orthotic exoskeleton, (**b**) lower limb prosthetic robot [1].

Figure 1 shows two examples of wearable robots. The scientific community differentiates exoskeletons from orthosis by defining the former as the devices that enhance the physical capabilities of wholesome users and the latter as the devices that assist persons with limb impairments [2]. Specifically, in Figure 1, the lower extremity of an orthotic exoskeleton for mobility problems is presented as developed by the authors, and the lower extremity of a prosthetic robot, according to work in [1], is presented in Figure 1b. Despite their differences, both devices act in parallel with the limb. In the medical field, in combination with rehabilitation therapies, exoskeletons can help patients with spinal cord injuries, strokes, and lower limb paralysis caused by hemiplegia [1].

The studies of the calculation of torque equations in each lower extremity exoskeleton joint were based on the kinematic analysis. Specifically, in [3], forward kinematics was applied to find the foot’s position when values were given for the corners of the joint. The torque required on each joint is determined using free-body diagrams of different joints. The work found in [4] proposed the lower limb robotic exoskeletons (LLRE) model. The free-body diagram of force on the knees and hips was constructed. Dynamic hip and knee models were obtained, considering the hips and knees as support points. The torque equations of the lower limb joints were calculated according to the parameters of the specific model. Another approach to calculating the joint torques was also based on kinematics. The kinematic analysis is applied through forward and inverse kinematics as proposed in [1]. The Euler–Lagrange method is used to obtain the dynamic equations of the exoskeleton. The literature review was performed by querying the Google Scholar database. To identify papers on robotic lower limb exoskeletons, we mainly focused on electric actuation technologies. The results were filtered based on the officially used torque calculation models to determine the percentages. Nearly 600 scientific articles have been published in the last three years on robotic exoskeletons for the lower extremities based on kinematics (thus, excluding the upper extremities cases).

Nearly 32% are surveyed on the topic, and 35% mainly present simulations of proposed models based on the formally used torque calculation models. The rest include works on reducing cost or power dissipation and applications of ML in the control of the exoskeleton. As the works above show, the researchers of robotic exoskeletons calculate the torques of the lower limb exoskeleton joints based on their model’s kinematic analysis.

It was noted by the authors that there is a lack in the international literature on the measurement of lower extremity joint torques embedding the physical part of the implementation, which differentiates significantly by both the building components of the exoskeleton and the user’s physical characteristics.

Thus, the following are issues that motivated this work, and the proposed approach to offer a combined solution is presented in the rest of this work.

1. Lack of a well-defined framework for calculating the torques of the joints based on multiple factors.
2. Creation of a set of parameters for simulating the operation and calculating the torques.
3. Inclusion of the user’s physical data in the calculation of the parameters for calculating the torques.
4. Use of the motor characteristics to assess their suitability or not for the targeted solution (exoskeleton).

Most authors seem to agree that clinical gait analysis (CGA) data sources are a good start for the initial design of the actuation to be used in their prototypes [5]. However, Beyl, in his work [6], remarks on the large variability observed in gait data and cautions designers of actuated exoskeletons to be careful in interpreting CGA data and formulating design recommendations based on those data. Joint torque data determine the required characteristics for the actuation to be applied at each assisted joint. The intensity of the joint torques fluctuates within the gait cycle [5,6,7,8], and therefore, in most cases, designers use maximum values (peaks) as requirements for the sizing of their actuators [5,6,7]. However, in [9], the authors used optimisation methods and models of human motion to estimate the required torques for their passive, assistive systems. The aforementioned shows the need for a method to optimise the torque calculation based on the characteristics of the human, the exoskeleton, and the motors selected to decrease the time-to-production and achieve smooth motion.

According to the above literature references, the mathematical model in this article differs from other models in terms of its variability. First, the user can configure multiple parameters that affect the robotic exoskeleton, taking into account the characteristics of the human body (weight, height, etc.) and the characteristics of the robotic exoskeleton (exoskeleton weight, actuator weight, etc.). Second, the proposed solution considers the self-correction of the model by allowing its dynamic modification.

**Figure 2:**
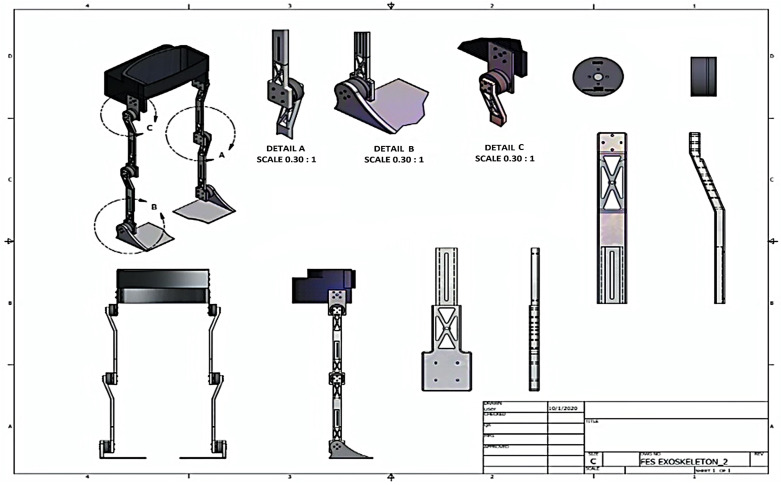
Robotic exoskeleton design plan of the Hybrid Exoskeleton FesRobex (Autocad design).

Figure 2 shows the robotic exoskeleton drawing showing the joints of the actuators. We are developing a hybrid rehabilitation system (FesRobex) combining Functional Electrical Stimulation (FES) and an exoskeleton to control patients’ gait with lower limb mobility problems. This study aims to use the mathematical model to calculate the torque at each joint and analyse the individual characteristics that influence the mathematical model for the appropriate selection of motors in exoskeleton joints.

First, in Section 1.1, an introduction to servo motors is offered, and Section 2 presents the proposed approach. Then, in Section 3, the evaluation of the proposed model and the calculations are offered, as well as its effectiveness in selecting the appropriate motors for embedding on a targeted exoskeleton. Finally, the paper concludes with Section 4 featuring the discussion and Section 5 outlining the conclusions of the mathematical model.

### 1.1. Introduction to Servo Motors

In this section, an introduction to servo motors that are used in the design and implementation of exoskeletons is provided. This part of the work is considered significant to highlight the variety of the available servo motors and emphasise the parameters that need to be considered when embedded in an exoskeleton.

#### 1.1.1. Research on Servo Motors

Servo motors have been used in automatic control systems for many years, especially in applications that require speed, position and torque control of the motor shaft. Classic examples include robotic arms, automatic machine tools, remote-controlled models, and automatic navigation systems for ships and aircraft [10].

**The essential characteristics of the servo motor**

- The motor torque is proportional to the applied control voltage that the amplifier develops due to the error at its input.
- The direction of the torque is determined by the control voltage’s polarity (instantaneous value).

Servo motors are structurally very similar to ordinary motors but are not identical. They differ because they contain measurement devices and a feedback system that is used in conjunction with a servo-drive mechanism to control torque, speed, or position [11].

#### 1.1.2. Criteria for the Selection of Servo Motors

The criteria for selecting servo motors are response speed, accuracy, and errors due to external distortions combined with the cost, availability, and reliability of the motor. Another important selection criterion is that the performance should cover both the power of the load (due) and the friction (losses) of the device. In addition, the servo motor must operate at the desired speeds and provide the required acceleration for the rotor and the load [12].

#### 1.1.3. Characteristics of the Servo Motor

*(a) Mechanical and geometric*

- The size, weight and inertia of the motor.
- The placement of the motor and the way it is connected to the moving mechanism.

*(b) Electromechanical*

- Required power and concentration of motor power (power to mass ratio).
- Torque requirements and characteristics.
- Speed range and response to changes.
- Sensitivity to changes in servo motor parameters [13].

#### 1.1.4. Features of Electric Servo Motors

- **DC Servo motors**

There are various types of electric servo motors, such as permanent magnet servo motors, rotor-controlled servo motors, stator-controlled servo motors, and stator and rotor servo motors in series.

- **R/C Servo motors**

**Figure 3:**
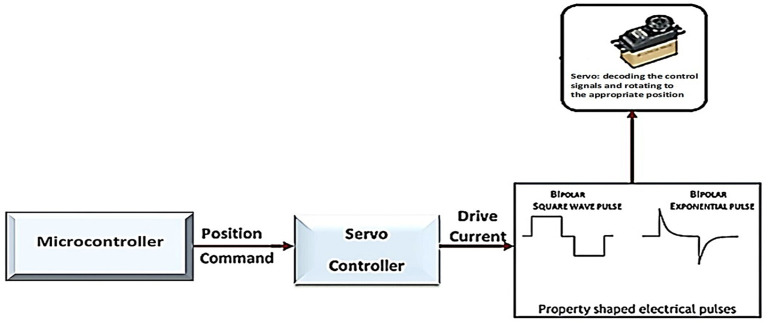
Operation RC Servo.

R/C servo motors are special compact servo motors that include a complete servo motor system consisting of a motor, a gearbox, a feedback device, and a drive and control circuit (Figure 3). The main parts constituting it are the following:

- DC electric motor;
- An electronic circuit controls the end drive shaft and gearbox position.

**Figure 4:**
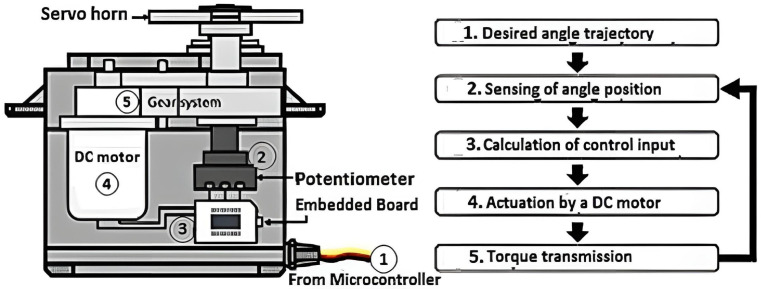
Schematic of an RC servo motor [14].

The final drive shaft does not perform complete rotations but rotates between two extreme positions. The servo operation requires (Figure 3) the provision of the appropriate electrical voltage and a signal that determines the position of rotation of the final shaft. The control of the servo requires a specialised controller, and the open-loop control method is used. The main disadvantage of RC servo motors is the inability to perform complete and continuous rotation (Figure 4).

Nevertheless, these servomechanisms have essential advantages such as:

- Low cost.
- Small dimensions and easy-to-use shape, which surrounds all parts.
- Produce high torque values.
- The use of sensors and feedback circuits is not required to determine the position of the driveshaft.

Servo motors are essential in robotics as they facilitate intelligent and natural movement. They are used in robotic systems of all kinds and can transmit information about the rotation of the motor on its axis so that the robot can “know” the movements of its various parts. The realisation of the desired movement of a robotic mechanism requires the combined movement of its joints. This is achieved by using a servo motor, which drives a mechanical system as a whole. In recent years, with the development of biomedical technology, servo motors have had broad applications in medicine and specifically in robotic medical systems [12].

### 1.2. Brushless and Brushed DC Motors

DC motors are used in applications where DC power sources are available, such as aircraft, automobiles or robotic systems. However, this type of small motor has some drawbacks. The main disadvantage is the excessive scintillation and wears on the brushes. Small and fast DC motors are too small to use compensation winding and auxiliary poles. The reinforcement reaction and the effects L $\frac{di}{dt}$ tend to create sparks in the transducer brushes. In addition, the high rotation speed of these motors increases the wear of the brushes, thus requiring shorter maintenance. In some applications, the maintenance required by the brushes of these DC motors may not be acceptable. A typical example is the DC motor in an artificial heart, where an incision must be made in the patient’s chest for maintenance. In addition, sparks in the brushes are dangerous, as they can cause an explosion and excessive RF noise. This developed a fast, small and reliable direct current motor with low noise and long life. This is a combination of a small motor that is very similar to a permanent stepper motor and has:

1. A cursor position sensor; and
2. An electronic circuit breaker.

These motors are called brushless DC motors (S.R.) because they are powered by a direct current source without switches and brushes. They are also called Modern Permanent Magnet Motors [15].

***The S.R. motors without brushes have many advantages such as:***

1. Size and low weight.
2. Relatively high performance.
3. Long service life and reliability.
4. Minimal or no maintenance.
5. Very low RF noise level compared to S.R. motors with brushes.
6. High-speed capability (50,000 rpm).
7. High torque.

Their main disadvantage is the high purchase cost.

Finally, in the introduction, we note that most studies use clinical gait analysis (CGA) data and human motion models to estimate the required torques for their passive support systems. As a result, there is no possibility of parameter variability (mass, length of body parts, height and weight of a person, the mass of actuators, etc.). Most studies use ready-made data from libraries and data they extract from their model with specific characteristics, as we mentioned in the introduction to the bibliographies. Therefore, they do not have the option to vary the characteristics of the robotic exoskeleton or the characteristics of the human body over a wide range to calculate the joint torque. The aim of the article is to fill the gap that has been created, i.e., the computation of torque with the possibility of changing parameters of the exoskeleton and human characteristics. The purpose of this work is to model the best choice of the appropriate motor (in the physical implementation phase) for each lower limb joint of the hybrid exoskeleton.

## 2. Materials and Methods

In this section, the materials of interest are initially presented along with their parameters. Then, the theoretical approach, according to the evidence from the literature, is explored, aiming at the comparative data for servo motors. Collecting the servo motors’ data is critical since introducing any servo motor to a robotic component dynamically affects the system’s characteristics, especially in the case of the inclusion of heterogeneous servo motors to various joints of the exoskeleton. The collected data are organised in a database to allow the selection of the appropriate motor for the targeted joint. The following two subsections refer to the parameters of the exoskeleton that should be considered for calculating the joints’ torques and the characteristics of the actuators that may be used. This concludes the extraction of the parameters and values of interest, developing the proposed mathematical model to calculate the torques of heterogeneous motors installed on an exoskeleton’s joints.

After the presentation of the materials and their characteristics, the methodology to select the appropriate motor for each joint follows. Next, the mathematical model is analysed, and the steps to be followed are presented. The proposed methodology has two steps. During initialisation, the parameters of the user are set based on the user’s characteristics (e.g., weight, height, etc.) and the coefficients derived from known models. In the first step (A), the masses for the exoskeleton are calculated, and the motors’ characteristics are considered as a penalty on masses. In the second step (B), the joint torque is calculated and verified with the desired one. If the achieved torque is sufficient, then the motor is selected. Otherwise, the process is repeated until an appropriate configuration is found.

### 2.1. Comparative Study of Servo Motors

In many automation control applications, the main competitors of servo motors are stepper motors. Both types of motors have their advantages and disadvantages. Their differences relate mainly to their performance because they are differently designed. For example, a rotor motor’s poles are much larger than the poles of a servo motor, so a rotation requires a larger current to flow through its windings. In addition, the stepper motor at high speeds degrades its torque, which is a phenomenon that can be reduced using a higher supply voltage. In contrast, the large number of poles of a stepper motor has a beneficial effect at lower speeds, thus giving it a torque advantage over a servo motor of the same size. Another difference is the way each type of motor is controlled. The open-loop method is used to control the stepper motors. This reduces the cost, as no feedback device is required (e.g., encoder for most positioning applications). However, in stepper motor systems, the excess power is converted into heat, thus generating a significant amount of heat in the motor and drive, which must be considered in various applications, especially those in the medical and healthcare field. Servo control solves this problem by supplying the motor with the current needed to move or hold the load. It can also provide maximum acceleration torque, which is often more significant than the maximum continuous torque of the motor. However, an encoder can also control a stepper motor in a complete closed-loop servo system. In terms of equipment, stepper motors are more superficial than servo motors.

Therefore, they are much easier to maintain and cost less, especially in small motor applications. When they operate within the design parameters, they do not lose their steps and do not require encoders. At the same time, when they are at rest, they remain stable, holding their position without any fluctuations, especially in dynamic loads. Specifically, a Brushless Direct Current (BLDC) generally operates better for speeds below 2000 RPM, lower acceleration values, and high retention torque. Servo motors are best in applications that require speeds above 2000 RPM and high torque at high speeds or where a high dynamic response is required. In conclusion, servo motor control systems respond better to high speeds and high torque applications involving dynamic load changes. BLDC motor control systems are less expensive than their respective servo motors and are ideal mainly for applications that require relatively low acceleration values, high holding torque, and flexibility to operate in an open or closed-loop system. For a complete picture of the differences between a Servo Motor SR (Brushed) and Step Motor (Hybrid) or BLDC motor, the table below shows the characteristics of a DC servo motor with a collector–brush system and a BLDC brushless motor (Table 1) [16].

We consider that the two motors are of the same quality and have the same rated power. There are many designs of robotic exoskeletons proposed for different purposes in the literature. Table 2 summarises some of the primary active projects in the scientific community with the types of motors they use, which have been reported in journals and conferences [1].

### 2.2. Robotic Exoskeleton Features

Exoskeletons are anthropomorphic mechanical devices worn by an operator that closely match the body’s anatomy and work in coordination with the user’s movements. Among the main requirements of an exoskeleton to be taken into account when designing are the following:

- **The design must be anthropomorphic:** Current designs have an abnormal shape; another limitation of exoskeletons is the lack of direct exchange of information between the human nervous system and the wearable robotic part.
- **The design must be flexible:** The length of the thigh, stem, and waist must be adjustable, and the variation in length and the stem is approximately 6 cm for average people, from 1.60 to 1.80 m. The length of the torso is approximately 0.246 times the height, and the length of the thigh is about 0.245 times the height.
- **Increase joint strength:** Exoskeletons do not transfer the substantial load to the ground but augment joint torque. This consideration might be used to reduce joint pain or increase joint strength in paralysed or weak joints.
- **Selection of Degrees of Freedom (DoF):** The exoskeleton must comply with the free movement of the joints. Table 3 shows the DoF of a lower extremity exoskeleton.
- **The exoskeleton robot actuator:** It must have a high output-to-weight power ratio and features such as low inertia, fast response, high accuracy, etc. [1].

The joints in the lower limb of the human body are the hips, knees, and ankles. Each joint has different abilities to move or DoF, as shown in more detail in Table 3. The types of lower limb exoskeletons based on joint motions are differentiated into several types based on how the actuators drive the exoskeleton. The actuators can drive just the hips, the knees, or the ankles.

In a small number of studies, exoskeletons have multiple actuators to drive a combination of joints. These combinations of actuators are hips and knees, knees and ankles, and all three joints (hips, knees, and ankles) [17].

### 2.3. Analysis of Actuators in Robotic Exoskeletons

Most types of actuators used in robotics cannot be used in exoskeletons since this application requires high speeds during operation at higher speeds than most actuators can provide. Electric, pneumatic, hydraulic, and Series Elastic Actuators (SEA) are the leading candidates available for use as actuators in exoskeletons. The design and selection of actuators was based on the average torque and power of each subject during normal walking (not pathological) at average speed. In addition, a study of different potential candidates was evaluated. The most relevant criteria for selecting activation technology for driving human joints were specific strength (activator power ratio to actuator weight) and portability. In this respect, linear hydraulic and pneumatic actuators have a high power density but are usually massive and present internal leakage and friction problems.

They have been used in some recovery devices but still face a standard limitation on the constant spring of the tyre element that is stable. Harmonic coordination of strength and position between patient and exoskeleton is complex between different subjects. The literature shows that the use of electric motors provides a reduction in energy consumption during walking. DC motors meet the criteria of the necessary power with a compact and portable solution for portable devices. Based on this, brushless DC motors connected to a harmonic drive gearbox were selected. Torque calculation is necessary to construct robotic exoskeletons and specialised robotic devices using servo and DC brushless motors [18].

### 2.4. Methodology of Selecting the Appropriate Motor

**Figure 5:**
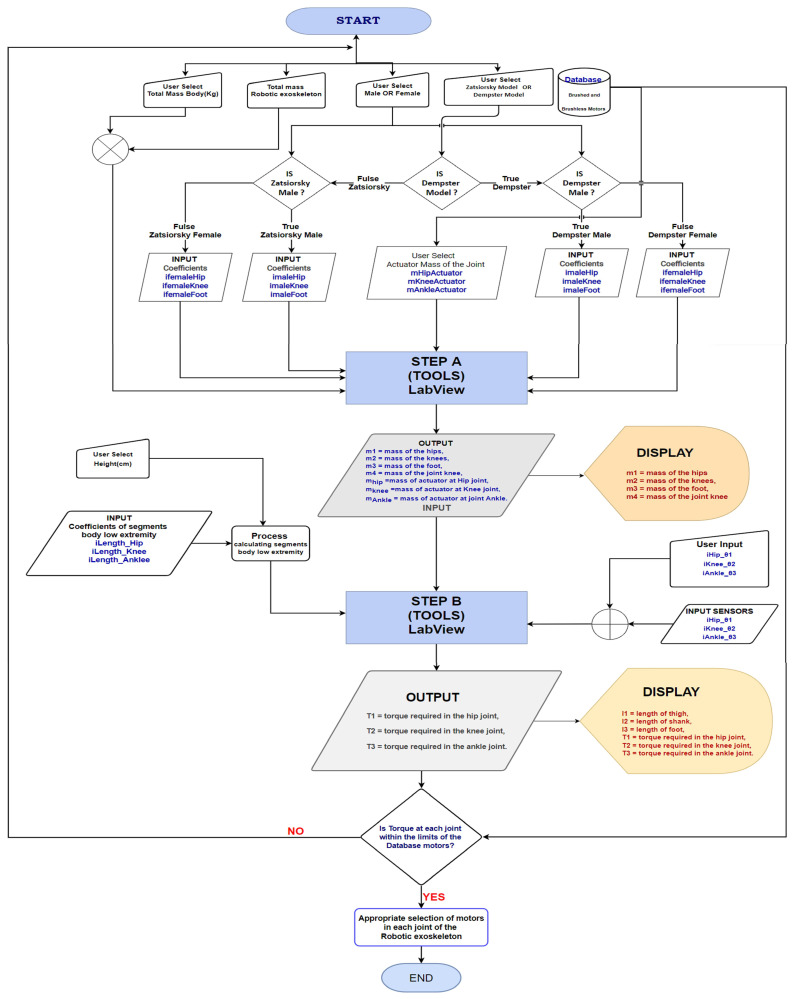
Flow chart Model.

As mentioned in this section, the proposed methodology comprises two steps. Initially, the characteristics of the user are set, and the parameters regarding the skeleton are set. Then, the coefficients are calculated based on known models, namely the Zatsiorsky BSP and the Dempster BSP (body segment parameters). In Step A, the masses of the exoskeleton’s parts are calculated, considering the collateral effect of installing a specific motor at a joint. This is a novel approach, since this effect was included (or not) in the mass of the body parts. The approach allows a more precise exploration of the motor under consideration, its effect on movement, and the motor’s verification (hence, selection) with the desired behaviour. In Step B, the torques are calculated using additional parameters set by the user or a group of sensors to calculate more precise data during kinematic analysis. At the end of this step, the torques are reported, and the motor is either characterised as appropriate, or the process is repeated from the start, rejecting the motor as unsuitable. Steps A and B have been implemented in the LabView for verification reasons and to facilitate researchers to implement these steps (see Figure 5, flow chart). The calculations include the extensive mathematical model and the database of DC motor characteristics. In this way, selecting the appropriate DC motor per articulation is automated. The implementation of the mathematical model for calculating lower extremity joint torques may be found on GitHub [19]. The fact guarantees the commonality of this methodology that it utilises the extended mathematical model, takes into account the characteristics of the available DC motors, takes into account their effect on the behaviour of the robotic exoskeleton and automates the selection of the appropriate DC motor at the joints’ lower extremity.

The calculations are based on the proposed extended mathematical model, which has as its fundamental principle that each lower limb robotic exoskeleton is an interconnected part of the human body. According to this assumption, every variable of the human body, such as weight, and height, affects the calculation of the mathematical model. Accordingly, the variables of the robotic exoskeleton, such as its total weight and the weight of actuators in the joints, should also be considered. It is essential to point out that the mathematical model can be used by any lower limb robotic exoskeleton researcher who wants to calculate the joint torques to choose correctly between brush and brushless DC motors at each joint. It has the ability to configure the parameters of the human body and corresponding variables of the robotic exoskeleton. It can also use a database of data on electric motors DC for robotic exoskeletons.

**Figure 6:**
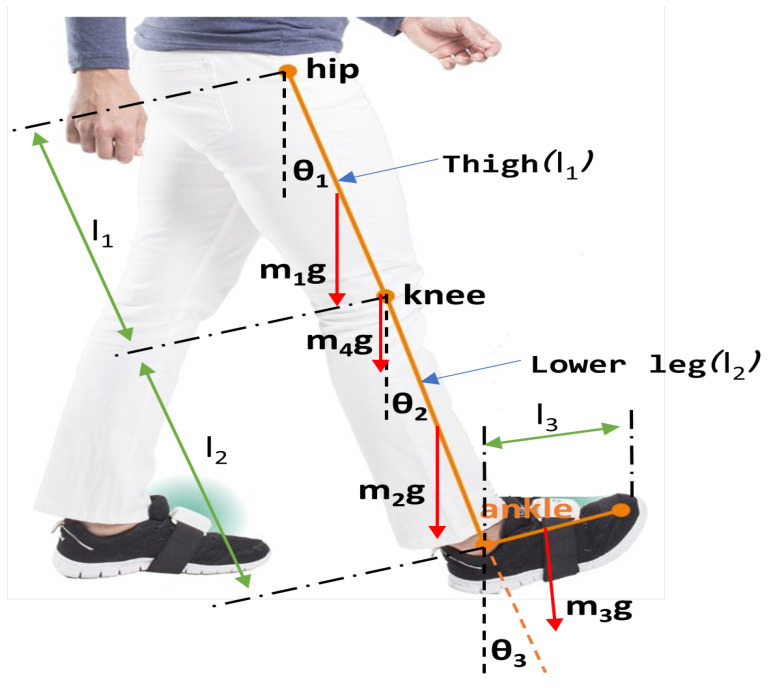
Force balance diagram of the lower leg.

**Figure 7:**
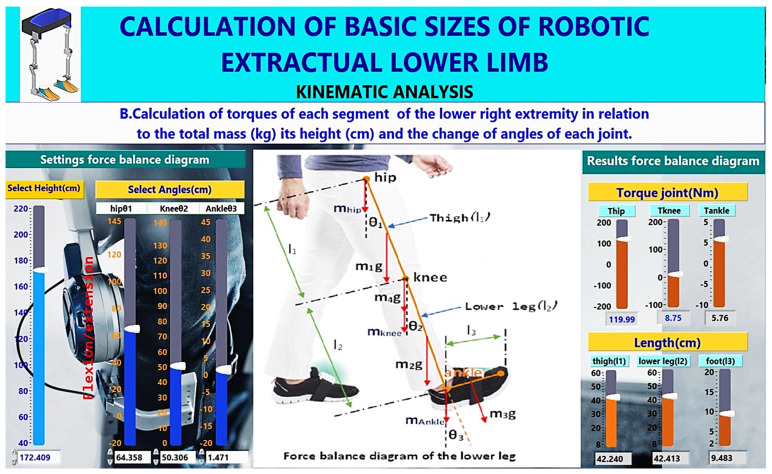
Calculation of the torques of each joint of the lower extremity.

In Section 2.4.1, the extended mathematical model is presented in detail regarding calculating the masses of the variables that affect each part’s mass, as shown in Figure 6. Then, in Step B of the methodology, as depicted in Figure 7, the calculation of the length of parts and torques of each part of the lower limb to the total mass (kg), its height (cm) and the change of the angles of each joint, as it is analysed in Section 2.4.2, is performed. The methodology allows the user to interact with a database containing characteristics of brush or brushless, as analysed in Section 2.4.3.

#### 2.4.1. Extended Mathematical Model for Calculation of Segment Masses, Centres of Mass

**Figure 8:**
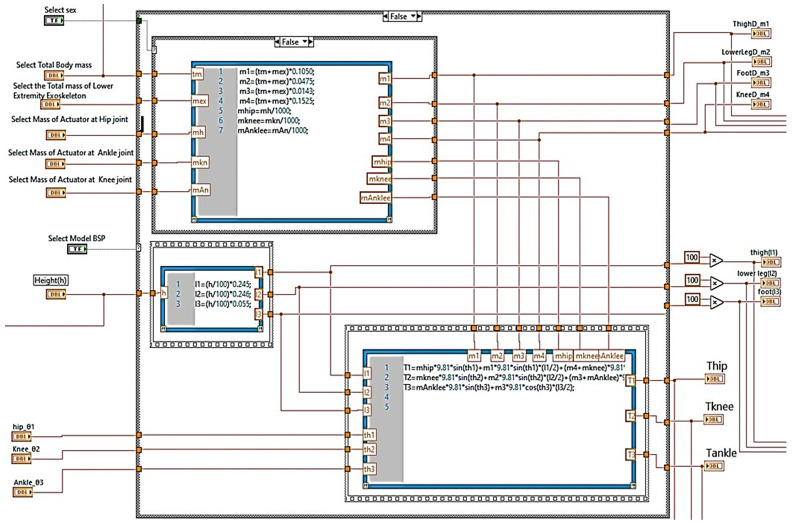
Calculation of Segment Masses, Centres of Mass and Length of Segment in LabView.

The mathematical model in Step A calculates the masses and centres of mass (COM) of the lower limb of the human body, such as the hip centre of mass (COM${}_{hip}$), the knee centre of mass (COM${}_{knee}$), the foot centre of mass (COM${}_{foot}$), and mass of the knee joint. The user can choose between two mass calculation models, the Zatsiorsky BSP and the Dempster BSP, and the gender (male or female), as seen in Figure 8, which is the Graphical User Interface (GUI) developed in LabVIEW.

Dempster’s method is reflected in Table 4, which gives the coefficients affecting body parts’ mass or centre mass. Cadaver data from Dempster (1955) are applied to water displacement data obtained from 135 living subjects (35 men and 100 women) to obtain the weight, centre of gravity, and radius of gyration for the segmented extremities. Some subjects (33 in total, 15 men and 18 women) were examined to obtain the weight of the segments of the trunk using the water displacement method, and 16 of these subjects (7 men and 9 women) were examined to locate the centre of gravity of each trunk segment [20]. In 1990, Zatsiorsky et al. determined the centre of mass for different human body segments. Each human body segment was divided according to the bony landmarks defined by Zatsiorsky. Data for this operation were collected via means of gamma-ray scanning, and the measurements were completed on 100 male and 15 female Caucasian subjects aged between 19 and 25 years old [21]. In 1993, de Leva observed that data provided by Zatsiorsky lead to many errors in the body COM calculation of USA college athletes. The source of those errors was caused by the body segmentation method, specifically by setting the reference points at bony landmarks. To obtain more precise results, de Leva decided to change the reference points from bony landmarks to the axis of rotation of body segments. To simulate the kinematic and dynamic behaviour of the body in movement, the body should be simulated. Segmentation methods allow the body to be modelled as connected segments are reduced to their centre of mass [22]. Zatsiorsky’s method is reflected in Table 5, where the coefficients affecting body parts’ mass or centre mass are given.

**Figure 9:**
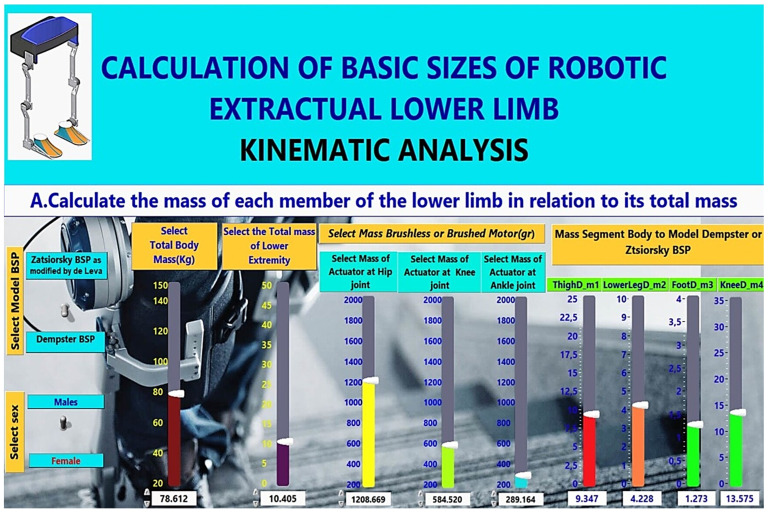
The mass of each part of the lower limb is calculated in relation to its total mass.

In addition, the user sets the total body mass in kg (see Figure 9). In Figure 8, the code for calculating the masses in LabVIEW is shown by applying the above coefficients to each calculation of mass and centres of mass of lower body parts (see Figure 7). Masses are calculated according to the following general relationship:

(1) $$m=t_{m}+m_{ex}\times Coef$$

t${}_{m}$ = Total mass body

m${}_{ex}$ = Total mass robotic exoskeleton - mass actuator (m${}_{actuator}$)

Coef = Coefficients affecting the mass or centre of mass of body parts.

**Figure 10:**
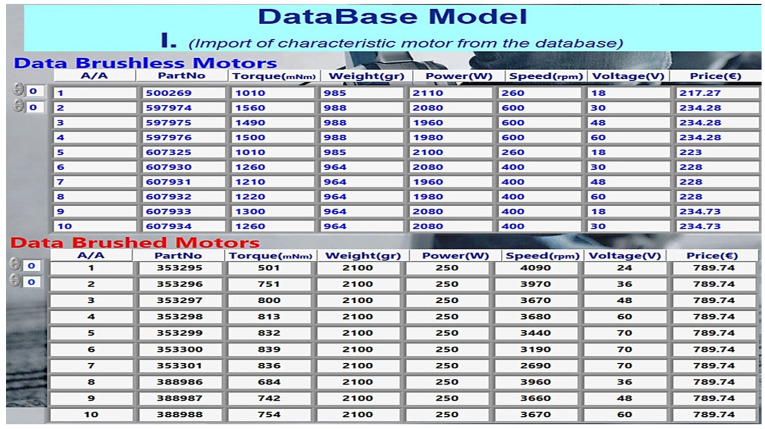
Characteristics of motors from database.

It is possible to adjust the mass of actuators at each lower limb joint (actuator mass selection at hip joint, actuator mass selection at knee joint, actuator mass selection at ankle joint) for each brushless motor or brushed motor (grams). The user in Figure 9 defines them according to the motor selection present in the database (see Figure 10).

Therefore, according to the above, the mass of the hip, the mass of the knee, the mass of the leg, the mass of the knee joint, the mass of the actuator at the hip joint, the mass of the actuator at the knee joint, and the mass of the actuator at the ankle joint are considered. They will then be used in the calculation of the torques that are analysed in the following section.

#### 2.4.2. Calculation of the Torque of Robotic Exoskeleton Joints

Figure 6 shows the Power Balance Chart of the lower leg, which shows the forces acting on the hip, knee, and ankle joints, that help determine the torque required on each joint. Using the robotic exoskeleton, the authors have designed and considered similar exoskeleton robots, such as robotic exoskeleton (LLRE) [4], and the following torque equations were calculated.

- **Hip torque calculation model equation:**

(2) $$\begin{array}{cc} T{}_{1}= & m{}_{hip}\times g\times sin\left(\theta {}_{1}\right)+m{}_{1}\times g\times sin\left(\theta {}_{1}\right)\times \left(l{}_{1}/2\right)+\left(m{}_{4}+m{}_{knee}\right)\times g\times sin\left(\theta {}_{1}\right)\times l{}_{1}+m{}_{2}\times \\ \; & g\times \left(sin\left(\theta {}_{1}\right)\times l{}_{1}+sin\left(\theta {}_{2}\right)\times l{}_{2}/2\right)+m{}_{3}\times g\times \left(sin\left(\theta {}_{1}\right)\times l{}_{1}+sin\left(\theta {}_{2}\right)\times l{}_{2}+cos\left(\theta {}_{3}\right)\times \left(l{}_{3}/2\right)\right)\\ \; & +\left(m{}_{3}+m{}_{Ankle}\right)\times g\times \left(sin\left(\theta {}_{1}\right)\times l{}_{1}+sin\left(\theta {}_{2}\right)\times l{}_{2}\right) \end{array}$$

- **Knee torque calculation model equation:**

(3) $$\begin{array}{cc} T{}_{2}= & m{}_{knee}\times g\times sin\left(\theta {}_{2}\right)+m{}_{2}\times g\times sin\left(\theta {}_{2}\right)\times \left(l{}_{2}/2\right)+\left(m{}_{3}+m{}_{Ankle}\right)\times g\times (sin\left(\theta {}_{1}\right)\times \left(l{}_{1}\right)+sin\left(\theta {}_{2}\right)\\ \; & \times \left(l{}_{2}\right))+m{}_{3}\times g\times \left(sin\left(\theta {}_{2}\right)\times l{}_{2}+cos\left(\theta {}_{3}\right)\times \left(l{}_{3}/2\right)\right) \end{array}$$

- **Ankle torque calculation model equation:**

(4) $$\begin{array}{c} T{}_{3}=m{}_{Ankle}\times g\times sin\left(\theta {}_{3}\right)+m{}_{3}\times g\times cos\left(\theta {}_{3}\right)\times \left(l{}_{3}/2\right) \end{array}$$

- **Parameters of calculations:**
  m${}_{1}$ = centre of mass of the hips;
  m${}_{2}$ = centre of mass of the knees,
  m${}_{3}$ = centre of mass of the foot;
  m${}_{4}$ = mass of the knee joint;
  m${}_{hip}$ = mass of actuator at hip joint;
  m${}_{knee}$ = mass of actuator at knee joint;
  m${}_{Ankle}$ = mass of actuator at ankle joint;
  g = 9.81m/$s^{2}$ gravitational acceleration;
  l${}_{1}$ = length of thigh;
  l${}_{2}$ = length of shank;
  l${}_{3}$ = length of foot;
  T${}_{1}$ = torque required in the hip joint;
  T${}_{2}$ = torque required in the knee joint;
  T${}_{3}$ = torque required in the ankle joint.

In Step B of the mathematical model, the torques at each joint of the lower limb are calculated, as shown in Figure 7, which is the GUI. In this step, the lengths of the lower limb body segments are calculated, selecting the person’s height. The user adjusts the angles (θ${}_{hip}$, θ${}_{knee}$, θ${}_{ankle}$) of the lower limb joints or with motion sensors. Since calculating all the above quantities, the mathematical model calculates the torques in each joint using the equations of motion mentioned above, which are integrated into the proposed mathematical model (see Figure 8).

#### 2.4.3. Reference Database

**Figure 11:**
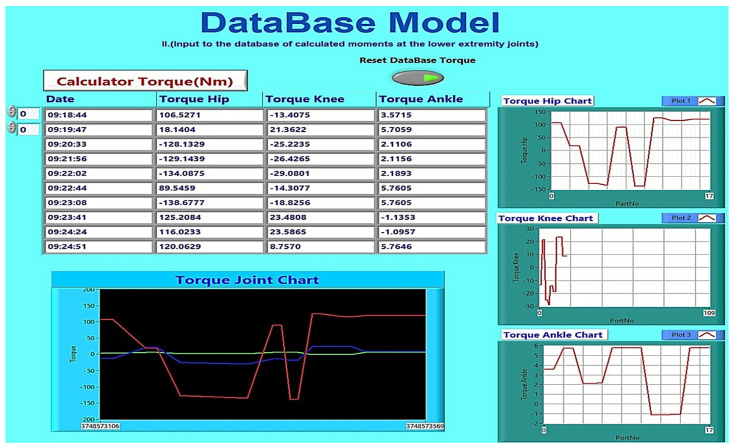
Joint torque calculations and SQL database storage (GUI).

The proposed methodology considers the characteristics of brushed and brushless motors from the database storing the motor characteristics. Figure 10 depicts the GUI of LabVIEW, in which the elements of the motors are entered in the panels brushless and brushed. The GUI allows collecting torque data (torque hip, torque knee, and torque ankle) and deleting and plotting the torques from the mathematical model, as shown in Figure 11. According to the data collected in the database, a researcher can choose the appropriate actuator needed in each joint of the robotic exoskeleton he will implement. It can also change every element in the mathematical model, as already mentioned in the flow chart of the model in Figure 5, and feedback of the robotic exoskeleton.

## 3. Results

Experimental measurements were performed for the proposed methodology, implementing it in LabView. Groups of males and females were considered, considering each individual’s height and weight parameters and the Dempster and Zatsiosky models, as shown in Table 4 and Table 5. All parameters are adjusted by the mathematical model as described before. According to the data collected, the following descriptive statistics for qualitative and quantitative characteristics were reported, exploiting the SPSS statistical analysis suite.

### 3.1. Quality Characteristics of Motors

Table 6 presents the quality characteristics of the brushless and brushless motors. The advantage of brushless over brushed is the greater lifetime, the high efficiency, the low inertia of the rotor, and the low electrical noise. Brushless motors have many positive quality features. The question is whether they cover the needs of the parameters (torque, weight) of the joints of the body’s lower extremities. The following subsection explores the use of the brushless over brushed motors by analysing the quantitative characteristics.

### 3.2. Quantitative Characteristics of Motors

Table 7 contains a comparison of brushless quantitative characteristics (BLDC) and brushed motors. The characteristics of brushless and brushed motors are from the Maxon library [24] and are registered in the database (SQL Server). Additionally, the user can add motor data from other company libraries to the database. This allows them to be compared with the data collected and processed in the mathematical model. In the descriptive statistics of Table 8, the maximum values of torques that can be used for each motor are found, allowing a numerical comparison. Finally, in the table, the results derived from the mathematical model in LabView are offered regarding the torques at each joint of the body’s lower limbs.

The mathematical model calculates the torques of the joints, the masses and the central masses of the parts of the lower extremities, as well as their lengths. It is essential that for the correct choice of motors in the joints, all the parameters related to the robotic exoskeleton must be taken into account: that is, the weight of the motors (entered in the mathematical model from the database) and the individual weights that are calculated by adjusting the weight of each body. This is the necessity of the descriptive statistics in Table 8 with the SPSS software in groups of men and women. The torques at each lower extremity joint through Table 8 give important information regarding the difference in motors selection at each joint, specifically a maximum torque at 145.75 Nm for males and 91.83 Nm for females, 34 Nm the knee for males and 20 Nm for females, and at the ankle 6 Nm for both groups.

The parameters of the mass and the centres of mass of the lower extremity parts of the body are essential. Therefore, they are also considered in calculating the extended mathematical model as a function of the body’s total mass and separate masses of parts. Furthermore, they are also considered in the calculation from the extended mathematical model as a function of the body’s total mass and separate masses of parts. Therefore, the descriptive statistics of the masses in Table 9 allow selecting the appropriate motors based on the torque and considering the weight of each joint of the body.

## 4. Discussion

Below, we mention methods of selecting electric actuators and calculating joint torques. Calanca et al. in [25], presented a methodology based on a graphical tool that matches the actuator’s capabilities with the task’s requirements. The proposed approach obtains the operating torques and speeds through experimental tests. A motion capture system allows positions and velocities to be acquired, while joint torques are calculated via inverse dynamics in a multi-body human exoskeleton model. Similarly, Barjuei et al. in [26], proposed an approach for selecting a brushless BLDC motor and a gearbox transmission based on optimisation through an analytical human–robot dynamic interaction model and a mathematical relationship between the weight and technical characteristics of its components. Finally, Belogusev and Egorov in [27] proposed an automatic measurement procedure for determining the starting torque of an electric gear actuator for an exoskeleton. In this work, a methodology is proposed for selecting the appropriate motor during the design phase, hence, at a higher abstraction level, avoiding experimental tests of the exoskeleton. Additionally, this work considered not only the characteristics of the human–robot interaction model but also the effect of candidate motors at each joint.

**Figure 12:**
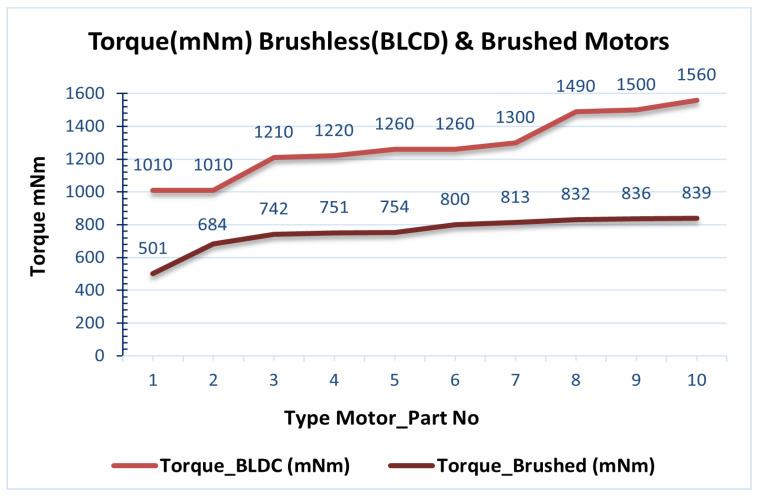
Comparison Torque (mNm) Brushless (BLDC) and Brushed Motor.

**Figure 13:**
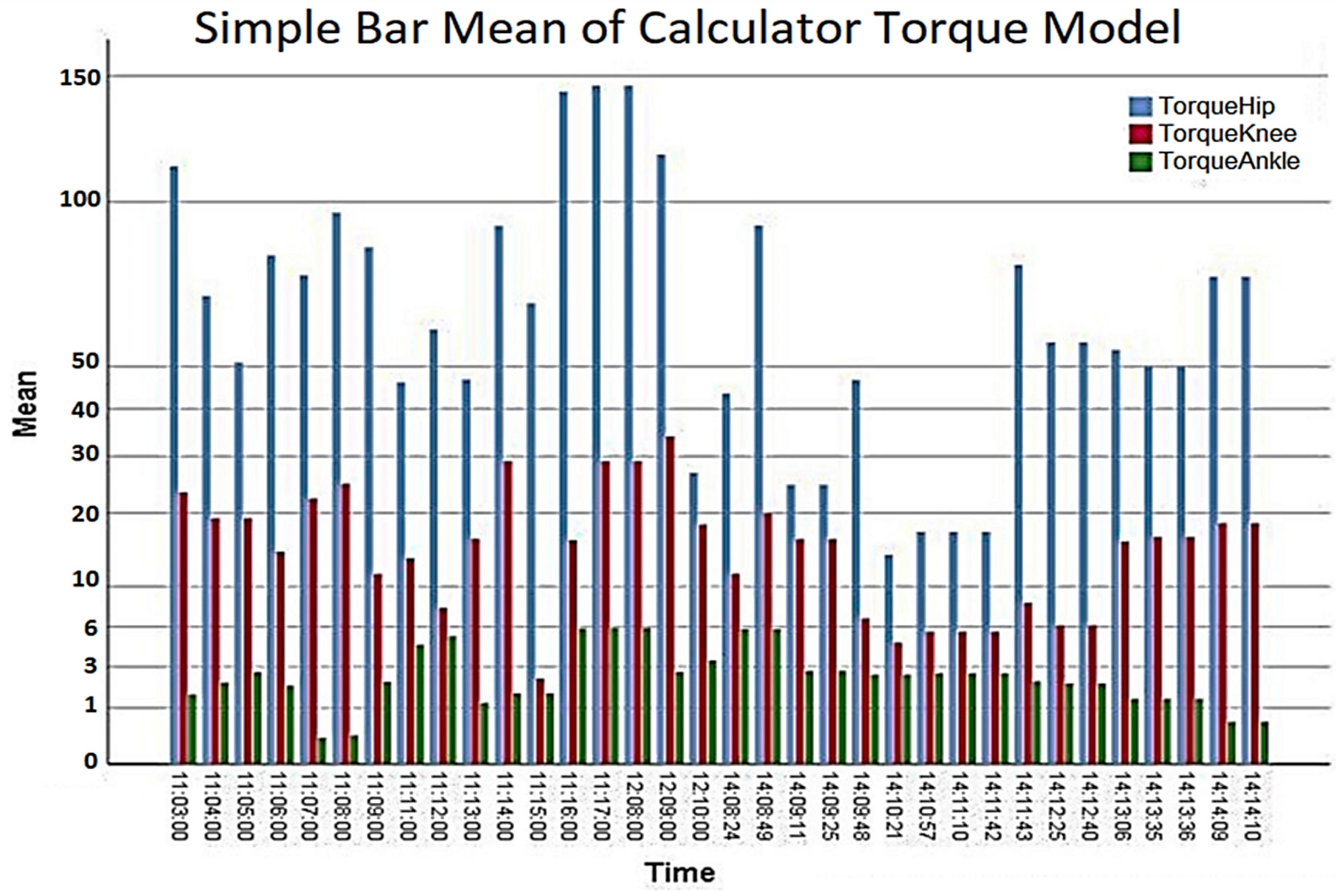
Simple Bar Mean of Torque Model Calculator.

In the results presented in Table 7, the maximum torques of brush and brush motors in mNm were obtained from the database, as shown in Figure 12. Furthermore, the graph in Figure 13 calculates torques at each joint at the lower end over time, according to the extracted parameters resulting from the mathematical model. Therefore, according to the previous data, a thigh motor is suitable if it offers a maximum torque of at least 150 Nm. Similarly, a knee motor is suitable if it offers a maximum torque of at least 35 Nm, and an ankle motor is suitable if it offers a maximum torque of at least 10 Nm.

Since the maximum torque range, as shown in Figure 13 (torques at each joint per time from the mathematical model), does not achieve the target torque, a gearbox (Harmonic Drive, CSD-20-160-2AGR [28]) is connected to the motor shaft to reduce speed and increase torque. So, at the peak torque from Figure 12, the 1560 mNm (1.56 Nm) brushless motors will be closer to the desired thigh torque values. A ratio of 160:1 gives each combination a continuous net torque of 71 Nm and peak torques of 180 Nm. Therefore, the average hip actuator torque of 71 Nm is considered sufficient for most patients.

The choice of brushless motors is an option because of the coverage of the maximum torque and other factors such as their weight. According to Table 7, there is less weight (1000 g) in brushless motors compared to brushed motors (2000 g). Conversely, choosing heavier motors will increase the overall weight of the robotic chassis. The weight of the robotic exoskeleton is critical to the system’s human factor stability, which highlights this work’s impact.

**Figure 14:**
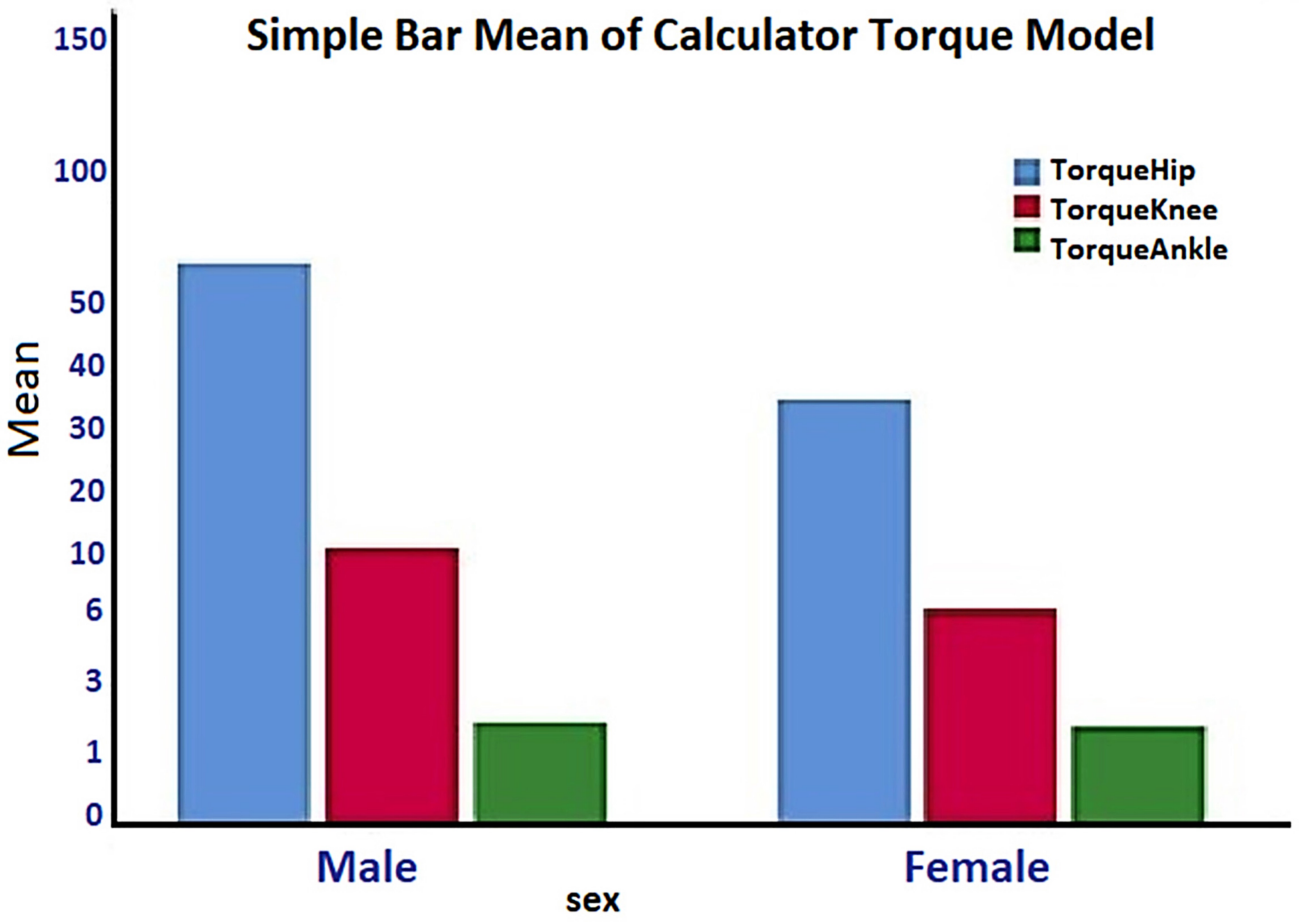
Simple Bar Mean of Calculated Torque—Male, Female.

In Figure 14 and Table 8 (Total torque), the calculation of the joints of the lower extremities in the male and women groups is shown. The torques at the knee and ankle joints have negligible differences in their maximum value between males and females, Tknee = 34 Nm and Tankle = 6 Nm. Thus, the motor required for the knee joint should perform a maximum torque Tknee = (34 + std. Deviation = 10N m) = 44 Nm. Likewise, the motor required for the ankle joint should perform a maximum torque Tankle = (6 Nm + std. Deviation = 2 Nm) = 8 Nm. According to Table 7, the torques of the joints in nominal torque (max. Continuous torque) = 1560 mNm or 1560 Nm.

The above calculations of the torques at the joints of the lower limbs of the human body and the calculation of the masses of the lower limbs and the masses of the actuator joint of the robotic exoskeleton, combined with the qualitative characteristics, were compared between brushless and brushed motors, as reported in Section 2.1. The following motor choices result in each joint of the robotic lower limb exoskeleton.

Initially, a choice of a motor (EC 90 flat Ø90 mm, brushless, 600 W [24]) for the thigh and knee was made at 15,600 mNm with the addition of a gearbox (Harmonic Drive [28], CSD-20-160-2AGR) connected to the motor shaft to reduce speed and increase torque. Regarding the ankle, the motor choice was based on a brushless one (EC 60 flat Ø60 mm, brushless, 100 Watt [29]) with the addition of a gearbox (Harmonic Drive [28], CSD-20-160-2AGR), which is connected to the motor shaft to reduce the speed and increase the torque, i.e., Tankle = (nominal torque, max. continuous torque = 227 mNm); (transmission ratio 160: 1) = 36Nm. Regarding the choice of the specific motors, their masses have been taken into account (1000 gr, brushless < 2000 gr, brushed) and nominal speed 1620 rpm (EC90), 3840 rpm (EC60) due to the use of a gear unit.

## 5. Conclusions

As we presented in the Introduction, the mathematical model proposed in this paper differs from other models in terms of its variability. As from the discussion above, the mathematical model has several positive elements. In the automated mathematical model, researchers for the lower limb robotic exoskeleton experiment with various parameters (centres of mass, common masses, total body weight, mechanical exoskeleton weight, actuator masses at each joint, human body height, and joint angles). Based on the mathematical model, the methodology calculates the torques at the joints of the lower limb and compares them with the motor database elements. So, it selects the appropriate motor at each joint.

Weaknesses in the mathematical model were also identified, such as the need to evaluate more parameters affecting the stability of the robotic exoskeleton. However, due to the uniqueness of the walking pattern, it is difficult for the lower limb exoskeleton robot to adapt to the different walking patterns of users. Therefore, embedding an AI model for walking patterns may be considered for future improvement. This will adjust the mathematical model based on the different walking patterns of the users and provide the appropriate motor selection in each joint for achieving the optimal solution.

## Figures and Tables

**Table 1:** Comparison of SR servo motor with brushes and hybrid stepper motor or BLDC motor.

Features	Servo Motor SR (Brushed)	Step Motor (Hybrid) or BLDC Motor
Cost	The cost of a servo motor or servo system is greater than the cost of a step motor system with the same rated power.	Step motors are generally cheaper than servo motors that have the same rated power value.
Size	They are available in a wide variety of sizes from small to very large motors and can operate huge machines.	In the step motors, there are not as many size options as in servo motors, especially for large sizes.
Noise	Servo motors produce very little noise compared to step motors.	They produce a small hum due to the screening process. However, a high-quality driving system reduces the level of this noise.
Power range	They are available in DC and AC motors and thus have a very wide range of available power.	The range of available power in step motors is smaller than in servo motors.
Performance	In general, servo motors are very efficient motors. In small loads mainly, a yield of 80–90% is reported.	The step motors consume enough power, a lot of which is converted into heat. Usually, their performance is at about 70% and depends on the driving system.
Life	Coolers every 2000 h of operation must be replaced. Codecs may also be needed to replace them	The only place that wears out is the bearing. This gives the step motors a slight lead.
Low speed high torque	They work very well in low-speed applications, mainly due to very low friction.	They provide more torque at low speeds (RPM).
High speed high torque	They maintain rated torque at about 90% of their speed without load.	Step motors lose up to 80% of their maximum torque, at 90% of their maximum speed.
Power ratio by weight/size	Given the effectiveness of the servo motors, they have an excellent power ratio in terms of weight and size.	Step motors are less efficint than servo motors. This usually means a lower motor size power–weight ratio.

**Table 2:** Robotic exoskeletons around the world.

Foundation	Name	Purpose	Technical Data	Control Method
Yonsei University South Korea	Help walking an exoskeleton of the lower extremity	Patients with lower limb paralysis	1. 200 W DC motor without brushes 2. Harmonic movements 3. Torques: Hip: 79.3 nm Knee: 42.2 nm	1. Active kinematic control 2. Stability check
Tsukuba University Japan	HAL3	Patients with lower limb paralysis	1. Servo Motor DC 2. Harmonic joint reducers	1. Conscious recognition based on plant pressure and torso angle
Automation Robotics Centre Hispania	ATLAS	Quadriplegic patients	1. Brushless motors (Maxon) 2. Harmonic reducers 3. Maximum torque 57 nm (hip)	1. CoP stability check 2. Conscious recognition
Berkeley Robotics and Human Engineering Laboratory USA	Lower extremity exoskeleton (BLEEX)	Allow personnel the ability to carry major loads such as food, rescue equipment, first-aid supplies	1.DC brushless motors 2.Harmonic reducers	1. CoP stability control 2. Conscious recognition

**Table 3:** DOF design for lower extremity exoskeleton.

Joints	DOF/ Movement	Freedom Range	Driving Power Required (N/m)
Hip	3/Flexion–extension	$-120^{\circ }/65^{\circ }$	80–100
Hip	3/Addition–abduction	$-30^{\circ }/40^{\circ }$	Spring
Hip	3/Rotation	$-30^{\circ }/30^{\circ }$	Spring
Knee	2/Flexion–extension	$-30^{\circ }/40^{\circ }$	45–70
Ankle	2/Pretonation–rotation	$-15^{\circ }/30^{\circ }$	Spring
Ankle	3/Dorsal bending–bending of the fingersn	$-20^{\circ }/50^{\circ }$	Spring
Ankle	3/Dorsal bending–bending of the fingersn	$-30^{\circ }/20^{\circ }$	Spring

**Table 4:** Body segment coefficients as calculated for Dempster model.

Dempster ${}^{b}$						
**Segment**	**Weight/Total Body** **Weight**		**Centre of Mass/** **Segment Length**		**Length Relative** **to Height**	
	**Female**	**Male**	**Female**	**Male**	**Female**	**Male**
Thigh	0.1050	0.1175	0.428	0.433	0.242	0.245
Knee	0.1525	0.1710				
Foot	0.0143	0.0133	0.500	0.500	0.151	0.152

*^b^* Based on Plagenhoef et al. (1983), based on Drillis and Contini (1966), based on Dempster et al. (1955) [23].

**Table 5:** Body segment coefficients as calculated for Zatsiorsky model.

	Zatsiorsky ${}^{a}$, as Modified by de Leva					
**Segment**	**Weight/Total Body** **Weight**		**Centre of Mass/** **Segment Length**		**Length Relative** **to Height**	
	**Female**	**Male**	**Female**	**Male**	**Female**	**Male**
Thigh	0.1478	0.1416	0.3612	0.4095	0.3685	0.422
Shank–Lower Leg	0.0481	0.0433	0.4416	0.4459	0.4323	0.434
knee	0.1849	0.1959				
Foot	0.0129	0.0137	0.4014	0.4415	0.2283	0.258

*^a^* Zatsiorsky et al. (1990), as modified by de Leva.

**Table 6:** Quality Characteristics of Brushless and Brushed Motors.

Commutation	BLDC Motor Electronic Commutation Based on Hall Sensors	Brushed Motor Brushed Commutation
Maintenance	Less	Periodic
Life	Longer	Shorter
Efficiency	High	Moderate
Rotor inertia	Low	Higher
Electric Noise	Low	Arcs in brushes generate noise
Control	Complex, expensive	Simple, inexpensive

**Table 7:** Comparison Quantitative Characteristics: Brushless (BLDC) and Brushed Motors.

Servo Motor		Min	Max	Mean	Std. Deviation
Brushless	Torque (mNm)	1010.00	1560.0	1282.00	190.42
(BLDC)	Weight (gr)	964.00	988.00	975.40	12.00
	Power (W)	269.00	600.00	432.00	128.60
	Speed (rpm)	1960.00	2110.00	2041.00	62.20
	Voltage (V)	18.00	60.00	36.00	16.70
	Price (€)	217.00	235.00	229.60	5.90
Brushed	Torque (mNm)	501.00	839.00	1282.00	190.42
	Weight (gr)	2100.00	2100.00	2100.00	0.00
	Power (W)	250.00	250.00	250.00	0.00
	Speed (rpm)	2960.00	4090.00	3602.00	414.75
	Voltage (V)	24.00	70.00	52.20	16.42
	Price (€)	790.00	790.00	789.74	0.00

**Table 8:** Report Torque (Nm).

Sex		Torque Hip	Torque Knee	Torque Ankle
Male	Mean	78.9534	19.2694	2.6254
	Std. Dev/tion	38.2399	9.5207	2.0741
	Minimum	7.2119	2.3286	0.1082
	Maximum	145.7430	33.9808	5.9167
Female	Mean	45.4926	11.5044	2.4046
	Std. Dev/tion	24.2209	5.6196	1.3984
	Minimum	13.8903	4.6761	0.5725
	Maximum	91.8309	19.9213	5.8193
Total	Mean	69.0198	16.9642	2.5598
	Std. Dev/tion	37.7666	9.2258	1.8904
	Minimum	7.2119	2.3286	0.1082
	Maximum	145.7430	33.9808	5.9167

**Table 9:** Report Mass and Centre Mass (Kg).

Sex		Total Mass	Thigh m1	Knee m4	Lower Leg m2	Foot m3
Male	Mean	79.141	11.697	15.504	3.806	3.806
	Std. Dev.	15.151	2.239	2.967	0.728	0.728
	Min	60.640	8.960	11.880	2.920	2.920
	Max	108.800	16.080	21.310	5.230	5.230
Female	Mean	54.214	7.154	9.561	2.407	2.407
	Std. Dev.	7.019	1.668	1.870	0.251	0.251
	Min	47.590	5.000	7.260	2.060	2.060
	Max	63.650	9.010	11.770	2.760	2.760
Total	Mean	66.021	9.306	12.376	3.070	3.070
	Std. Dev.	17.034	3.008	3.867	0.884	0.884
	Min	47.590	5.000	7.260	2.060	2.060
	Max	108.800	16.080	21.310	5.230	5.230
